# Effects of Vitamin C on Growth and Non-Specific Immune Response of *Labeo gonius* Fry in Density-Dependent Biofloc Rearing

**DOI:** 10.1155/2024/9930544

**Published:** 2024-10-12

**Authors:** Ng Chinglembi Devi, Gusheinzed Waikhom, Soibam Khogen Singh, Pronob Das, Sourabh Debbarma, Reshmi Debbarma, Lukram Sushil Singh, Martina Meinam, Pradyut Biswas, Surajkumar Irungbam

**Affiliations:** ^1^Department of Aquaculture, College of Fisheries, Central Agricultural University, Agartala 799210, Tripura, India; ^2^Krishi Vigyan Kendra, ICAR Research Complex for NEH Region Manipur Centre, Ukhrul 795142, Manipur, India; ^3^ICAR-Central Inland Fisheries Research Institute, Regional Centre, Guwahati 781006, Assam, India; ^4^Fisheries College and Research Institute, Tamil Nadu Dr. J. Jayalalithaa Fisheries University, Thoothukudi 628 008, Tamil Nadu, India; ^5^Department of Fish Genetics and Reproduction, College of Fisheries, Central Agricultural University, Agartala 799210, Tripura, India

**Keywords:** biofloc, health status, stocking densities, vit C, water quality

## Abstract

Biofloc technology offers a viable choice for the rearing of fish seed by offering a safe and protected habitat for young fish. Nevertheless, it is crucial to establish a standardised stocking density and implement effective ameliorative steps in order to successfully utilise this technology. In this study, a 90-day investigation was conducted to assess the effect of stocking density and dietary vitamin C (Vit C) levels on the growth and immunity of *Labeo gonius* fry (1.03 ± 0.01 g) reared in a biofloc system. Three stocking density groups (5, 10 and 15 fish per 50 L) were established, and each experimental group was supplemented with three levels of Vit C (0, 500 and 1000 mg kg^−1^). The highest survival rate was observed in the lowest density group (five fish per 50 L) fed with 1000 mg kg^−1^ Vit C. A better feed conversion ratio and significantly higher protein efficiency ratio were found in the moderate stocking density group (10 fish per 50 L) fed with 500 mg kg^−1^ Vit C. Total leukocyte count (TLC), haemoglobin, and packed cell volume improved in Vit C-fed groups. The total erythrocyte count (TEC) increased in groups fed Vit C and stocked at lower densities (5 and 10 fish per 50 L). Total serum protein (TPP) content increased when Vit C was added at a rate of 500 mg kg^−1^. Serum glucose and cortisol levels were significantly reduced in Vit C-supplemented groups. Supplementation of Vit C at 500 mg kg^−1^ resulted in a significantly lower value of malondialdehyde (MDA). Thus, the findings confirm that the incorporation of Vit C in the basal diet promotes the growth and health status of *L. gonius* fry reared in the biofloc system at high-density rearing.

## 1. Introduction

Indian aquaculture is progressing at a fast pace, and carp farming forms the main backbone of the total freshwater aquaculture sector. The fry production business forms a promising entrepreneurial option to sustain livelihood security, and the country currently produces more than 52 billion fish advanced fry as of 2019–20 [1]. Earlier, traditional fry production in outdoor ponds was harnessed to supply advanced fry for culture practice; however, concerns about land availability, the need for supplementary feeding and other factors such as tadpole infestation and eutrophication were the few drawbacks and challenges faced [2]. To address these challenges, biofloc technology is a system that gives the scope of economic as well as environmental sustainability with intensification in production. It works by stimulating heterotrophic bacterial growth in the system by adding carbohydrates. The heterotrophic bacteria present in the system take up nitrogen, thus reducing the ammonium concentration. In contrast to conventional water quality control techniques, biofloc technology offers a sustainable, economical and easy-to-implement alternative. Low dependence on water makes it a promising alternative for aquaculture production in temperate and urban areas. The biofloc technology is useful in maintaining optimum water quality parameters under a zero-water exchange system, thus, preventing eutrophication and effluent discharge into the surrounding environment [3, 4]. The technology supports the supply of good-quality advanced fry by improving the reproductive performance of aquaculture animals and by enhancing larval immunity and robustness [5–8]. Moreover, the technology has proven useful in ensuring biosecurity, as there is limited water exchange except for sludge removal.

Success in any aquaculture system depends on the growth and survivability of the species. This can be achieved by maintaining proper stocking density. Therefore, optimisation of the stocking density of the cultured fish is very essential. Stress due to increased stocking density has deleterious effects on growth, feed utilisation, anti-oxidant and immune systems, welfare and health of cultures and species [9–14].Therefore, alleviating the adverse effects of stress and/or strengthening immunity are important goals of the aquaculture industry. It is observed that biofloc systems support the supply of good-quality advanced fry by improving the reproductive performance of aquaculture animals and by enhancing larval immunity and robustness [5–7]. Vitamin C (Vit C) is a crucial micronutrient vitamin that is involved in a number of physiological functions. It functions as an anti-oxidant to neutralise free radicals like reactive oxygen species (ROS) and reactive nitrogen species, protecting cellular constituents from damage caused by radicals while promoting growth, non-specific immunity and immunity against infections [15–17], environmental stressors and toxins [18]. As a nutritional intervention, incorporation of Vit C in the diet of the fish is required for proper functioning of the body as it is an essential micronutrient that cannot be synthesised by the experiment fish due to a lack of the enzyme L-gluconolactone oxidase [19]. Vit C studies in fish have also been focused on the possibilities to reduce negative impacts caused by stress and environmental factors upon health and disease resistance. As several studies reported that, Vit C supplements had a major impact on the immune system, improved growth development, survival and performance in fishes [15, 20]; hence, it is a fine immunomodulator candidate due to its ameoriolative effects.

*Labeo gonius* known as Kuria Labeo is an indigenous minor carp of India under *Cyprinidae* family which has been identified as an important species for diversification of aquaculture practices, especially in north-eastern states of India [21–23]. The growth of this species is a little slower compared to rohu and catla. However, they are easily marketable at 100–300 g as compared to 700–800 g for Indian major carps. It is a highly fecund fish (1.5−2 lakh/kg) like rohu and is hardy in nature as compared to other cultivable carp species [22, 23]. The species has also been found to have high consumer preference due to its good taste and is believed to have high medicinal properties. Much work on fry rearing and grow-out culture of fish in biofloc system is reported. However, very limited work is done on the fry rearing of minor carps in the biofloc system. It is quite evident that stress due to an increased stocking density has deleterious effects on growth, feed utilisation, anti-oxidant and immune systems, welfare and health of cultures and species [9–14], and thus, a nutritional biotechnology approach for biofloc system at high-density rearing can harness its improvisation.

Considering these backdrops and the importance of this minor carp species in terms of economics, nutrition and species diversification in aquaculture, the present study attempted to evaluate the effect of stocking density and Vit C on growth, survival and physiological alterations under high-density fry rearing of the *L. gonius* and the amelioration capacity of Vit C in the biofloc system. To the author's knowledge, no work has been initiated towards fry rearing of carps in biofloc system and the role of stress ameliorating agents in high-density rearing. The findings from this preliminary study can generate a precise knowledge on the species suitability and methods to improve growth and survival in a biofloc system.

## 2. Materials and Methods

### 2.1. Experimental Fish and Rearing Conditions

**Figure 1:**
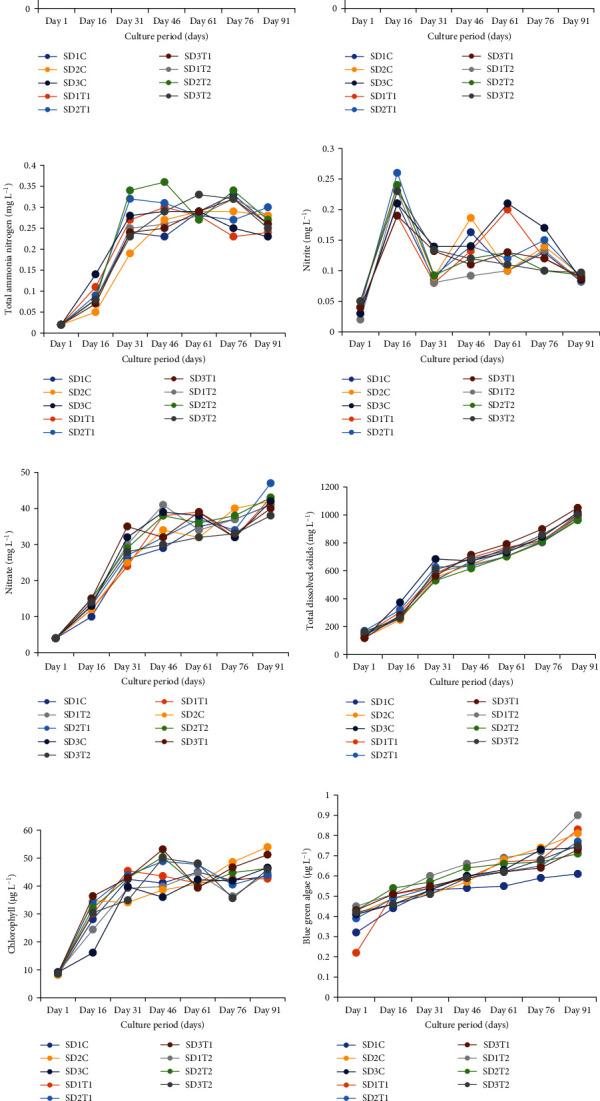
Water quality parameters of all treatments throughout the experiment such as (A) temperature (°C), (B) pH, (C) disolved oxygen (mg L^−1^), (D) alkalinity (mg L^−1^), (E) total ammonia-nitrogen (mg L^−1^), (F) nitrite (mg L^−1^), (G) nitrate (mg L^−1^), (H) total dissolved solids (mg L^−1^), (I) chlorophyll (µg L^−1^), (J) blue green algae (µg L^−1^), (K) specific conductivity (µs cm^−1^), (L) floc volume (mL L^−1^).

Advanced fry of *L. gonius* (1.03 ± 0.01 g) was collected from the tank-based fry-rearing facility of the College of Fisheries, Central Agricultural University, Lembucherra, Tripura, India, to the indoor experimental tanks. The work is approved by the Institutional Animal Ethics Committee (IAEC) of the College of Fisheries, Central Agricultural University (CAU), Imphal, Tripura vide office number CAU-CF/48/IAEC/2018/10. Due care and animal welfare measures of the system were taken strictly as per guidelines. The fish were kept in well-aerated tanks for 1 week prior to the experiment and were fed commercial feed containing 32% protein (CPF India Private Limited) during this period. The water temperature, dissolved oxygen (DO), total dissolved solids (TDS), chlorophyll content, blue green algae content and specific conductivity was measured using EXO1 Multiparameter sonde (Yellow Springs Instrument Company, Ohio, USA; Product Code: 599501–00), while pH was checked using a pH metre-335 (Systronics). The alkalinity was checked by titrimetric method [24]. Total ammonia-nitrogen (TAN) was estimated spectrophotometrically using phenate method (630 nm), while nitrite-N and nitrate-N at 543 nm as per APHA [24]. The optical density in all cases was read with a UV-visible Spectrophotometer (UV1800- pharmaspcc, Shimadzu). To maintain uniform water quality parameters in the system, monitoring of water quality was done every day throughout the experiment. The maintained range is given in Figure 1A–L.

### 2.2. Experimental Design

The experimental fish were randomly selected and assigned to 27 tanks with 80 L capacities. Using a 3 × 3 factorial design, three stocking density groups (5, 10 and 15 nos. fish in 50 L^−1^of water) were established and each experimental group was fed with three levels of Vit C (0, 500 and 1000 mg kg^−1^) for 90 days.

### 2.3. Floc Preparation and Maintenance

Floc preparation was done 2 weeks prior to the start of the experiment applying our previous method [25]. Water was filled up to 25 L in a closed drum and an aeration facility was supplied. Liming was done so as to maintain the alkalinity level. As the water used was 25 L, the liming dose was 1.5 g in 25 L (@600 kg/ha). Twenty-five grams of raw salt was added for microbial growth stimulation. A commercially available molasses (Speciality Sugars Limited, Dhampur Dist Bijnor, UP- 246761; 43% carbon) was used as a carbon source. Fish feed was added as a nitrogen source and maintained C:N ratio at 20:1. If the total amount of ammonia was found to be 1 mg L^−1^ in 4 tons water (1 ton = 1000 L). That implies the tank water contains 4000 mg or 4 g of ammonia, that means the total average nitrogen content in ammonia was 82%. Thus, the total nitrogen content in tank water is 3.3 g. For eliminating 3.3 g of nitrogen, 66 g (20 times) of organic carbon is required. Carbon sources like molasses contain 43% carbon. Therefore, 153 g of molasses is to be added to the tank water after fermenting it for 24 h. Commercial probiotic ECO-BAC (CPF India Private Limited), composition: Bacillus Species (10^8^ CFU g^−1^) was added for bacterial growth. A pinch of yeast was added to elevate the process. Floc volume was checked every day to ensure uniform floc availability in the experimental tanks.

### 2.4. Experimental Feed and Feeding

Experimental feed was prepared by addition of Vit C (L-ascorbic acid, HiMedia Pvt Ltd., India) at amount of 0, 500 and 1000 mg kg^−1^, respectively, by spraying over commercial feed which is containing 32% protein (CPF India Private Limited). The formulation of commercial feed was consist of protein, 30%–32%; fat, 4%–5%; fibre, 7%–8%; moisture, 11%; meat meal, 33%; soybean meal (min), 4% and rice bran, 5.5%; whereas the composition of commercial feed was composed of fish meal, soybean meal, rice bran, broken rice, fish oil, rapeseed meal, vitamins and minerals.

After spraying, the experimental feeds were air-dried for 6 h at room temperature (25°C). In order to immobilise Vit C, the experimental feeds were coated with 4% aqueous gelatine in distilled water at a ratio of 5:40 (v/w) as a binder [26]. In the control diet, the same amount of gelatine was used. The experimental feed was stored in the freezer at 4°C until use. Hand feeding was done two times a day (09:30 and 16:30) until satiation.

### 2.5. Sampling Procedure

After the trial, three fish were harvested from each individual tank (*n* = 3) using a hand net. They were given mild anaesthesia (clove oil at 50 μg mL^−1^) immediately. Blood was drawn carefully from the caudal peduncle region with a 1 mL hypodermal syringe (24-gauge needle) which was previously rinsed with EDTA (2.7%) solution. Blood samples were pooled and transferred to the EDTA coated test tubes and shaken well to avoid blood cell haemolysis. For the serum collection, blood was collected in the Eppendorf tubes without EDTA, and allowed to clot for 2 h, which was then centrifuged (3000 x *g* for 15 min).

### 2.6. Fish Growth and Survivability

To monitor the growth performance of the fish, following parameters were used to evaluate the growth performance:

i. Mean weight gain (g)= Final weight (g)– initial weight (g),
ii. Weight gain (%) :  Weight gain/initial weight × 100,
iii. Specific growth rate (SGR; %/d)=ln[(Final weight)–ln(initial weight)] × 100/experimental period in days),
iv. Survival percentage (%) = Total number of harvested fish/total number of initial stock × 100,
  v. Fulton's condition factor K= Final weight gFinal length cm3×100.

### 2.7. Feed Utilisation Performance

i. Apparent feed conversion ratio (FCR)= Feed given (dry weight)/body weight gain (wet weight),
ii. Apparent protein efficiency ratio (PER)=Body weight gain (wet weight)/crude protein fed.

### 2.8. Blood and Immune Biochemistry

Estimation of total haemoglobin (g%) was done by using Sahli's haemometer. The haematocrit (Ht) value of blood samples was measured following the method of Schaperclaus, Kulow and Schreckenbach [27]. Total erythrocyte count (TEC) and total leukocyte count (TLC) were determined using a haemocytometer and observed under a microscope. Blood was diluted in Hayem's solution (Qualigens, India) for TEC and Turk's solution for TLC. Total serum protein (TPP) and albumin in serum was measured using a diagnostic kit (DIATEK Healthcare Pvt. Ltd., Kolkata, India) based on biuret method [28]. Globulin (G) was calculated by subtracting albumin values from serum total protein [29]. For the determination of glucose in serum, glucose diagnostic kit (Coral Clinical Systems, India) was used which is based on GOD/POD method [30]. The cortisol analysis is done by the ELISA Kit (CALBIOTECH Cortisol ELISA CO368S). Production of oxygen radicals from phagocytes in blood was measured using nitro blue tetrazolium (NBT) dye as described by Anderson and Siwiki [31]. The lysozyme activity of serum was determined following a turbidimetric method [32], modified to a microtitre plate assay.

### 2.9. Anti-Oxidant Enzyme Assay

Anti-Oxidant enzymes such as superoxide dismutase (SOD), glutathione-S-Transferase (GST) and malondialdehyde (MDA) were assessed.

The enzyme SOD activity was assayed according to the method of Misra and Fridovich [33] based on the oxidation of epinephrine and renochrome transition by the enzyme. To 0.1 mL of the sample, 1.5 mL of carbonate and bicarbonate buffer was added. The reaction was initiated by the addition of 0.4 mL of epinephrine and measured the change in optical density per min at 480 nm for 2 min in a UV spectrophotometer. One unit of SOD activity is the amount of protein required to give 50% inhibition of epinephrine auto-oxidation.

GST was determined by the method of Habig, Pabst and Jakoby [34]. The method is based on the principle of formation of an adduct of CDNB, S-2, 4-dinitrophenyl glutathione. The S-2, 4- dinitrophenyl glutathione (CDNB) is used as a substrate.

The enzyme MDA were assayed following the method of Hodges et al. [35]. The fresh weight of gill was taken and homogenised separately 1 mL of 20% w/v trichloroacetic acid (TCA) without thiobarbituric acid (TBA) and 20% w/v TCA containing 0.25% TBA and heated at 95°C for 25 min, cooled and centrifuged at 14,000 rpm for 10 min. Absorbance of the supernatant was recorded at 440, 532 and 600 nm and MDA content was calculated as an index of lipid peroxidation.

### 2.10. Liver Function Test Parameters

Glutamate-oxaloacetate transaminase (GOT) and glutamate pyruvate transaminase (GPT) were assessed to check the liver health. Determination of GOT and GPT activity was carried out using serum glutamate-oxaloacetate transaminase (SGOT) and serum glutamate pyruvate transaminase (SGPT) diagnostic kit (Diatek Healthcare Pvt. Ltd., Kolkata, India) based on UV-Kinetic method [36].

### 2.11. Statistical Analysis

The data obtained were analysed statistically and interpreted by using suitable statistical method with Statistical Package for Social Sciences (SPSS, version 23.0 for windows). One-way ANOVA was performed to determine the differences between the mean values of different treatments and two-way ANOVA was performed to study the interactions between different stocking densities and different Vit C concentrations. Duncan's new multiple regression test was used to compare the differences between the means at *p*  < 0.05. The data are represented as mean ± standard error (S.E.) in all cases.

## 3. Results

### 3.1. Growth Performance and Feed Utilisation

Growth performance and feed utilisation of *L. gonius* reared in different stocking densities are shown in Table 1. Survival rate was found to be significantly increased (*p*  < 0.05) in Vit C treated groups as compared to control. Lowest survival was found in the control of highest stocking density group (15 nos. 50 L^−1^) and highest survival rate was found in lowest density group (five nos. 50 L^−1^) treated with 1000 mg kg^−1^ Vit C. Higher stocking density significantly reduces the growth rate (*p*  < 0.05). Highest weight gain was found in lowest stocking density group (five nos. 50 L^−1^) treated with 500 mg kg^−1^ Vit C. And the lowest growth rate was found in control (0 mg Vit C) of highest stocking density group (15 nos. 50 L^−1^). Vit C treated group showed higher growth rate as compared to control. Treatments with 500 mg kg^−1^ Vit C supplementation showed higher growth rate as compared to control and 1000 mg kg^−1^ Vit C treated groups. Feed conversion ratio (FCR) was observed to be gradually reduced in Vit C treated groups with different doses. Significantly highest (*p*  < 0.05) FCR was found in control of highest stocking density group (15 nos. 50 L^−1^) and significantly lowest (*p*  < 0.05) FCR was found in the moderate stocking density group (10 nos. 50 L^−1^) treated with 500 mg kg^−1^ Vit C. Among all groups, protein efficiency ratio (PER) was found to be significantly highest (*p*  < 0.05) in the moderate stocking density group (10 nos. 50 L^−1^) treated with 500 mg kg^−1^ Vit C and significantly lowest (*p*  < 0.05) was found in control of highest stocking density group (15 nos. 50 L^−1^).

### 3.2. Effect on Haematological Parameter

**Figure 2:**
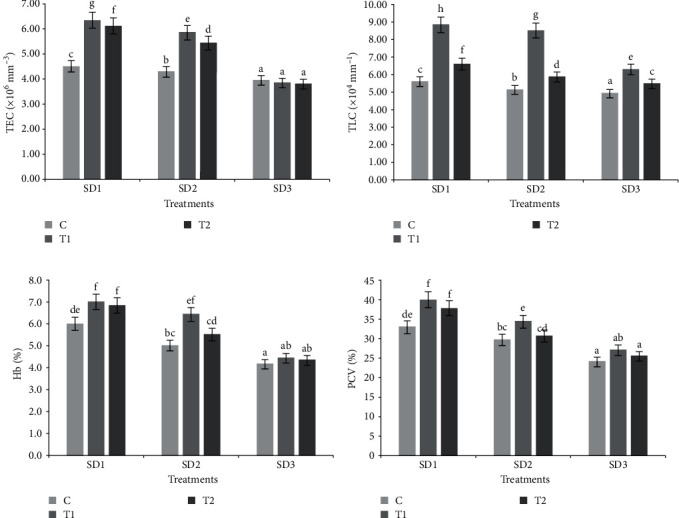
(A–D) Haematological parameters of *L. gonius* reared at three different stocking densities and fed vitamin supplemented diets at different doses.

The observed changes in blood haematology in groups are shown in Figure 2 and Table 2. TEC was found to be increased in lower stocking density groups (5 and 10 nos. 50 L^−1^) when treated with Vit C; however, no significant change was seen in highest stocking density groups (15 nos. 50 L^−1^) even after incorporation of Vit C. Total leucocyte count was found to be increased in all the stocking density groups when Vit C was incorporated. Highest TLC value was found in lowest stocking density group (five nos. 50 L^−1^) treated with 500 mg kg^−1^ Vit C, and lowest TLC was found in the control of highest stocking density group (15 nos. 50 L^−1^). Haemoglobin was found to be increased when Vit C was incorporated in the treatments. Highest haemoglobin was found in lowest stocking density group (five nos. 50 L^−1^) treated with 500 mg kg^−1^ and 1000 mg kg^−1^ Vit C and lowest haemoglobin content was found in control of highest stocking density group (15 nos. 50 L^−1^). Packed cell volume (PCV) also showed an increase trend when Vit C was incorporated. Highest percent of PCV was found in Vit C treated group of lower stocking densities (5 and 10 nos. 50 L^−1^) and lowest percent of PCV was found in the control and 1000 mg kg^−1^ Vit C treated group of highest stocking density (15 nos. 50 L^−1^).

### 3.3. Effect on Immunological Parameter

Significant increase (*p*  < 0.05) of TPP can be seen in the lowest stocking density group (five nos. 50 L^−1^) when Vit C was added at the rate of 500 and 1000 mg kg^−1^. However, in the other higher stocking density groups (10 and 15 nos. 50 L^−1^), significant increase of TPP was seen only when Vit C was added at the rate of 500 mg kg^−1^. Albumin content also showed significant increase (p  < 0.05) when Vit C was added at the rate of 500 and 1000 mg kg^−1^ in the lowest stocking density group (five nos. 50 L^−1^). However, no significant change in albumin was seen in higher stocking density groups (10 and 15 nos. 50 L^−1^) after the addition of Vit C. G as well as albumin and globulin (AG) ratio does not show any significant change (*p*  > 0.05) as compared to the controls in any of the treatments. Lysozyme activity was found to be increased when Vit C (500 and 1000 mg kg^−1^) was incorporated in all the treatments. Respiratory burst activity was found to show significant decrease in highest stocking density group (15 nos. 50 L^−1^) supplemented with 1000 mg kg^−1^ Vit C (Table 3).

### 3.4. Effect on Stress Parameter

Serum glucose and serum cortisol was significantly found to be the highest (*p*  < 0.05) in control of highest stocking density group (15 nos. 50 L^−1^) and it was found to be significantly reduced (*p*  < 0.05) when Vit C was supplemented (Table 4). SGOT and SGPT did not show any significant change in the treatments. GST was found to increase with increase in stocking density however Vit C supplementation significantly reduces the level. SOD levels were found to be almost similar in all the treatments. MDA increases significantly with increase in stocking density. Supplementation of Vit C at the rate of 500 mg kg^−1^ gives significantly lower value of MDA as compared to supplementation of Vit C at the rate of 1000 mg kg^−1^.

## 4. Discussion

### 4.1. Effects on Growth and Feed Efficiency

A clear understanding of the performance of a new species in biofloc system in terms of its adaptation potential and maximum utilisation of the biofloc eco-environment must be considered as first step in technology validation. The study demonstrates a clear influence of increased stocking density on the body growth of *L. gonius* fry. Increasing stocking density decreased the growth in terms of weight gain and specific growth rate (SGR). The possibility of chronic stress conditions and subsequent demand for energy to cope up with the situation might reduce the growth rate and food utilisation. This process mostly involves a catabolic pathway and that ultimately mobilise energy reserves [14, 37]. These observations coincide with earlier reports by Zaki et al. [38] in Nile Tilapia, where reduced growth of the fishes at high density was noticed. The improved growth at lowest density is possibly a result of better water quality, alongside lesser competition for space. As evidenced earlier, the biofloc environment supposedly contribute to maintaining a good water quality, alongside supply of floc protein to sustain an increasing fish density. Unlikely, the higher density group experienced severe crowding stress [39] in this study. In few other cases, higher density performs well in biofloc based nursery rearing like that of African Catfish, *Clarias gariepinus* [40]. In another study, a negative effect of increased stocking density on final weight and survival of Nile tilapia (*Oreochromis niloticus*) in biofloc system was reported by Lima et al. [41]. While in other studies, decreased weight gain was observed in *Piaractus brachypomus* when the stocking was increased from 20 to 40 m^−3^ [42]. These observations suggest that the robustness and adaptation capacity of the fish species in nursery biofloc is also influenced by the species type, mostly its hardiness and food extraction capacity. Alternatively, we approached with the amelioration capacity of Vit C to the induced crowding stress, if any. The observed data suggest potential of the Vit C at 500 mg kg^−1^ feed to effectively improve the growth of the fish. The protective effect of dietary Vit C in fishes has been well established [43, 44]. The growth potentiation is mostly related to the improvement in the immune capacity resulting from the multiple biological properties of the supplemented Vit C that regulate anti-oxidant, and anti-inflammatory activities and mediate cell death pathways [45]. However, a higher dose did not work well in our study, compared to the lower dose elucidating optimal requirement in stress challenge. As regards to the feed utilisation (FCR and PER), this study indicates a better efficiency in the moderate stocking density (10 nos. 50 L^−1^), compared to lowest stocking density (five nos. 50 L^−1^) and highest stocking density (15 nos. 50 L^−1^). The consistent supply of floc proteins along with the effective floc grazing capacity of the fish must have contributed towards the food supply, which curtails the supplementary feed supply in our study. In support of this study, Dawood, Koshio and Esteban [46] reported that an appropriate dose of Vit C helps improve feed intake and feed utilisation of aquatic animals, which directly influence the growth performance. Furthermore, the dietary Vit C can act as a cofactor during hydroxylation of lysine and proline, and lysine which prompts the protein synthesis that manifest an improvement in fish biomass gain [47]. Similar observations are reported earlier by Zaki et al. [38] in Nile tilapia. Under normal rearing conditions, an optimal Vit C supplementation in few commercial aquaculture species like yellow catfish (114.5 mg kg^−1^) [48]; yellow drum, *Nibeaalbi flora* (142.2 mg kg^−1^) [49]; golden pompano, *Trachinotus ovatus* (49.73 mg kg^−1^) [50]; largemouth bass (102.6 mg kg^−1^) [51] and Nile tilapia, *O. niloticus* (400 mg kg^−1^) [52] manifest better growth and feed utilisation. However, the need to support the animal in the face of overcrowding in biofloc system as discussed here needs proper evaluation. Moreover, it is noteworthy to highlight that a perfect comparison of stocking density effects among reported studies are difficult due to the variations in species and the culture system adopted.

### 4.2. Effect on Non-Specific Immune Response

Blood biochemical profile can precisely indicate the general health well-being and may deviate due to both nutritional and environmental disturbances [29, 53]. In this study, nonvitamin-fed biofloc groups experienced a decline in the haematological values at the highest stocking density (15 nos. 50 L^−1^), while lower density group (5 and 10 nos. 50 L^−1^) showed significant improvement in their indices. This corresponds to earlier observations in Nile tilapia where decreased levels of these blood indices (TEC, TLC, hemoglobin (Hb) and hematocrit (Hct)) were noticed at higher stocking density in biofloc set-up [38, 54, 55]. While, the supplementation of Vit C at 500 mg kg^−1^ feed was found to improve the blood profile significantly in lower density group, although highest density groups did not improve in haematological profile. This suggests a dose-dependent positive effect of dietary Vit C in the fish reared in biofloc system. The improvement in the TEC is probably a direct influence of the provided anti-oxidant vitamin in the amendment of the erythrocyte membrane against the produced free radicals in the crowding stress situation [56].

Moreover, Vit C is known to participate in erythropoiesis via stabilisation of the haem group during the synthesis of haemoglobin and thus, preventing the formation of methaemoglobin that can cause erythrocytic haemolysis. Therefore, the combining effect of the anti-oxidant [57], similarly, increases in TEC in Vit C (500 mg kg^−1^) delineates the immune-stimulating effect as observed here. A reduced level of Hb and Ht value in the highest density group (nonsupplemented) suggests compromised immune capacity in the fish. While, the supplemented groups showed plausible improvement in the profile that corroborates the immune-stimulating and stress-ameliorating function of Vit C [58].

The immunological profile explained by the total protein, AG levels showed the direct influence of stocking density induced stress and vitamin supplementation. A robust innate-immune cascade is linked with a rise in proteins, mostly AG, in the fish serum [59]. Proteins are used to maintain homeostasis, detoxification and tissue repair [58]. Therefore, it can be altered by environmental stress. In this study, there was no significant change in the G content which was similar to the findings of Yarahmadi et al. [60] where stocking density did not cause any significant change in the albumin levels. However, the total protein and albumin levels showed a plausible increase in the supplemented group at 500 and 1000 mg kg^−1^ in the lowest density group. And in the moderate and highest stocking density group, an increase in the total protein and albumin levels were seen in the 500 mg kg^−1^ supplemented group. This implies that Vit C was helpful in inducing a strong immune response in higher density. For the nonsupplemented group, we could not draw whether the biofloc which is composed of probiotic bacterial aggregate was able to contribute to immune enhancement [61, 62], as because we did not run a control group in this work.

Both lysozyme and respiratory burst activity are also vital constituents of fish immunity. Lysozyme is an antimicrobial peptide released by the leucocyte possessing a bactericidal effect through the lysis of the bacterial cell wall [63]. The supplementation of Vit C at 500 and 1000 mg kg^−1^ showed an improvement in response in all the stocking densities. It is established that Vit C has a strong immune modulating capacity which includes cell proliferation, macrophage infiltration, natural killer cell (NKC) activity, complement activity, lysozymes levels, phagocytic activity of leucocytes, development of cytokines and antibody concentrations [64]. Montero et al. [65] also reported elevation of serum lysozyme activity at high stocking density except in fish fed the vitamin (C and E) supplemented diets.

Respiratory burst activity is determined by the NBT assay which explains the ability of phagocytic cells to produce oxygen radicals [66]. In the present study, a gradual increase in respiratory burst activity can be seen with the increase in stocking densities suggesting that more phagocytic activity was there due to the increase in stocking density. Gatta et al. [67] reported a significant increase in phagocytic activity of rainbow trout, *Onchorhynchus mykiss* when exposed to stress exerted by chromium. However, no significant change in NBT was seen in lowest and moderate stocking density when Vit C was added but reduce in NBT can be seen in highest stocking density when Vit C was added at the rate of 1000 mg kg^−1^ showing the ability of Vit C at a higher dose to reduce the oxygen radicals when fishes are stocked at higher density in biofloc system.

### 4.3. Effect on Anti-Oxidant Enzyme Status

In the face of stress challenge such as high-stocking fish farming, the free radicals and ROS are continuously generated. As a natural defence, aquatic animals harbour key enzymatic anti-oxidant such as SOD, GST and MDA to eliminate these harmful radicles [48]. The enzyme, SOD helps maintain the free radical balance and protect the tissues and cells from oxidative stress [68]. Superoxide anions are converted to H_2_O_2_ by SOD, as a first defence mechanism for oxygen toxicity [69]. In this study, no significant change in SOD in all the treatments were observed which may be due to the metabolites converting effect of biofloc.

Glutathione is one of the important components in cellular anti-oxidant systems as it acts as a detoxifying agent for endogenous radical species, and also it is important in enzymatic detoxification reactions as a co-factor [70]. GST catalyses the linkage between xenobiotics or ROS and GSH to produce xenobiotic metabolites for excretion [69]. In our experiment, the elevation of GST level can be seen with the increase in stocking density possibly due to the increase of ROS with subsequent increase in SD. However, supplementation of Vit C at both doses significantly reduces the GST level in the higher stocking densities.

On the other hand, MDA can signify the level of lipid peroxidation and oxidative stress in the host [71]. In the present work, an increasing trend in the levels MDA in nonsupplemented groups was noticed explaining the stress likeliness due to overcrowding. Similar to the present study, Long et al. [72] also found an increase in MDA in the higher stocking density group of juvenile Chinese sturgeon, *Acipenser sinensis* in a recirculating aquaculture system. However, the Vit C supplemented groups showed a better anti-oxidant system at a supplementation dose of 500 mg kg^−1^, while the dose of 1000 mg kg^−1^ did not exert much effect as 500 mg kg^−1^ in our study. This evidence articulate that provided Vit C can act as a powerful anti-oxidant which agrees with earlier reports [73]. It is precisely explained that Vit C possesses the properties to scavenge free radicals, and therefore, protect the cell membranes and sulfhydryl groups of some enzymes from oxidative damage [74].

### 4.4. Effect on Glucose and Cortisol

Glucose and cortisol are the major indicators of stress. Catecholamine secreted by fish due to stress increases the conversion of liver glycogen to blood glucose to meet the energy demand by increased cell metabolism due to the induced stress [21]. In this study, increase in stocking density caused a significant increase in glucose level of *L. gonius*; however, supplementation of Vit C could protect the change in levels in the highest stocking density. It is stated by Barros et al. [57] that an increase in serum glucose concentration is a compensatory regulation in vertebrates for the higher energy demand under stress situations faced by the fishes.

Cortisol levels in fish blood can directly indicate the deleterious effects of toxic substance exposure [75]. Sanderson et al. [76] also reported a significant increase in cortisol of rainbow trout, *Onchorhynchus mykiss* exposed to ammonia. In the present study, a significant increase in serum cortisol was observed in the highest stocking density group indicating there was stress when stocked at 15 nos. 50 L^−1^. Similar to our findings, Montero et al. [65] reported higher cortisol level in higher stocking densities suggesting that the fishes were not able to adapt to that condition. Yarahmadi et al. [60] also reported significant increase in glucose and cortisol in higher density compared with lower density. However, supplementation of Vit C does reduce the cortisol level significantly showing the stress-reducing effect of Vit C.

### 4.5. Effect on Hepatic Functions

Alkaline phosphatase enters the blood after cell necrosis in tissues [77]. Therefore, SGOT, SGPT, and alkaline phosphatase (ALP) is used to assess the damage in the liver and kidney. Elevated enzymatic serum components are considered widely as reliable and sensitive indicators of cellular damage [78]. In our present study, SGOT, and SGPT of *L. gonius* were found to have no significant difference in all the treatments suggesting that there were no liver damages due to the effect of stocking densities and Vit C supplementation. Increase in GOT and GPT was reported by Agrahari, Pandey and Gopal [77] in *Channa punctatus* (Bloch) due to environmental stress. Similar findings to the present study were reported by Tewary and Patra [79], where level of GOT and GPT were not significantly different in their study indicating that Vit C does not affect GOT and GPT levels to a large extent.

## 5. Conclusion

The study's overall findings indicate that the biofloc system is highly suited for rearing *L. gonius* fry. When determining the ideal stocking level, it may be inferred that lower stocking density promotes to better growth in the biofloc system compared to higher stocking density. The alleviated immunological capacity of the fish and the resulting stress caused by high density were observed. Nevertheless, the addition of 500 mg kg^−1^ of dietary Vit C could successfully alleviate the stress, leading to better growth and survival of *L. gonius* fry in the biofloc system. Therefore, this work broadens the current paradigm of growing prospective carp species using biofloc, within a diversification programme. Moreover, Vit C plays a crucial role in reducing stress and can be effectively used as a supplement in high-density fish growth within a biofloc system.

## Figures and Tables

**Table 1:** Effects of stocking density and dietary vitamin C on growth performance and feed utilization in *Labeo gonius* reared in biofloc system.

Treatments		Parameters						
Stocking density (nos. 50 L^−1^)	Vit C (mg kg^−1^)	Weight gain (g)	Weight gain (%)	SGR (%/d)	AFCR	APER	Survival rate (%)	Fulton's condition factor
5	0	6.87 ± 0.17^d^	671 ± 17.60^d^	2.27 ± 0.025^d^	2.16 ± 0.29^cd^	1.19 ± 0.22^ab^	66.7 ± 17.6^ab^	1.2 ± 0.02^a^
	500	10.49 ± 0.10^h^	1032 ± 8.35^h^	2.7 ± 0.008^h^	1.31 ± 0.09^ab^	2.11 ± 0.20^de^	73.3 ± 13.3^ab^	1.46 ± 0.13^b^
	1000	7.87 ± 0.16^f^	774 ± 16.70^f^	2.41 ± 0.021^f^	1.49 ± 0.03^ab^	1.87 ± 0.04^cd^	100 ± 0^b^	2.12 ± 0.15^c^

10	0	5.98 ± 0.09^b^	591 ± 11.12^b^	2.15 ± 0.017^b^	1.88 ± 0.17^bc^	1.51 ± 0.16^bc^	70 ± 11.5^ab^	1 ± 0.08^a^
	500	8.94 ± 0.06^g^	880 ± 1.78^g^	2.54 ± 0.002^g^	1.2 ± 0.02^a^	2.46 ± 0.04^e^	86.7 ± 3.33^b^	1.16 ± 0.09^a^
	1000	7.32 ± 0.08^e^	720 ± 4.23^e^	2.34 ± 0.005^e^	1.43 ± 0.04^ab^	2.04 ± 0.06^d^	86.7 ± 3.33^b^	1.06 ± 0.04^a^

15	0	5.42 ± 0.06^a^	530 ± 5.95^a^	2.04 ± 0.010^a^	2.73 ± 0.47^e^	1.00 ± 0.17^a^	48.9 ± 16^a^	1.09 ± 0.03^a^
	500	7.26 ± 0.09^e^	716 ± 8.60^e^	2.33 ± 0.01^e^	1.47 ± 0.03^ab^	2.03 ± 0.06^d^	82.2 ± 8.01^ab^	0.97 ± 0.06^a^
	1000	6.46 ± 0.07^c^	633 ± 7.07^c^	2.21 ± 0.01^c^	1.62 ± 0.08^abc^	1.84 ± 0.10^cd^	88.9 ± 8.01^b^	1.11 ± 0.04^a^

**Two-way ANOVA: *p* values**								

Stocking density		<0.05	<0.05	<0.05	<0.05	<0.05	>0.05	<0.05
Vitamin C		<0.05	<0.05	<0.05	<0.05	<0.05	<0.05	<0.05
Interaction		<0.05	<0.05	<0.05	>0.05	>0.05	>0.05	<0.05

*Note: n* = 3. Overall mean value having different superscript in the same column shows a significant difference (*p* < 0.05; mean ± S.E.); alphabets denote the significant differences between different treatments.

**Table 2:** Two-way ANOVA of the haematological parameters of *L. gonius* cultured for 90 days.

Stocking density (nos. 50 L^−1^)	Vitamin C (mg kg^−1^)	TEC (×10^5^)	TLC (×10^4^)	Hb (%)	PCV (%)
**Two-way ANOVA: *p* values**					

Stocking density	—	<0.05	<0.05	<0.05	<0.05
Vitamin C	—	<0.05	<0.05	<0.05	<0.05
Interaction	—	<0.05	<0.05	>0.05	>0.05

*Note: n* = 3. Overall mean value having different superscript in the same column shows a significant difference (*p* < 0.05; mean ± S.E.); alphabets denote the significant differences between different treatments.

Abbreviation: Hb, hemoglobin.

**Table 3:** Effects of stocking density and dietary vitamin C on immunological parameters in *Labeo gonius* reared in biofloc system.

Treatments		Parameters							
Stocking density (nos. 50 L^−1^)	Vit C (mg kg^−1^)	Lysozyme (Unit min^−1^ mL^−1^)		NBT (OD 540)	TPC (g dL^−1^)		Albumin (g dL^−1^)	Globulin (g dL^−1^)	A/G ratio
5	0	25.71 ± 1.18^a^		0.481 ± 0.01^a^	3.36 ± 0.09^abc^		2.44 ± 0.09^abc^	0.92 ± 0.16^a^	2.85 ± 0.51^a^
	500	27.76 ± 0.31^b^		0.463 ± 0.02^a^	3.90 ± 0.14^d^		2.84 ± 0.15^c^	1.07 ± 0.16^a^	2.84 ± 0.61^a^
	1000	29.52 ± 0.77^c^		0.482 ± 0.06^a^	3.76 ± 0.26^cd^		2.74 ± 0.08^bc^	1.01 ± 0.21^a^	2.91 ± 0.49^a^

10	0	26.56 ± 0.65^ab^		0.599 ± 0.03^b^	3.39 ± 0.11^abc^		2.37 ± 0.03^ab^	1.02 ± 0.13^a^	2.43 ± 0.38^a^
	500	32.31 ± 0.93^d^		0.610 ± 0.01^b^	3.67 ± 0.02^bcd^		2.52 ± 0.35^abc^	1.16 ± 0.35^a^	3.33 ± 1.89^a^
	1000	31.33 ± 1.11^d^		0.615 ± 0.03^b^	3.41 ± 0.05^abc^		2.44 ± 0.02^abc^	0.97 ± 0.05^a^	2.53 ± 0.15^a^

15	0	26.2 ± 1.10^ab^		0.922 ± 0.07^c^	3.09 ± 0.21^a^		2.07 ± 0.02^a^	1.02 ± 0.19^a^	2.19 ± 0.45^a^
	500	31.31 ± 1.39^d^		0.895 ± 0.01^c^	3.22 ± 0.04^ab^		2.20 ± 0.06^a^	1.02 ± 0.10^a^	2.22 ± 0.30^a^
	1000	31.54 ± 0.54^d^		0.553 ± 0.01^ab^	3.11 ± 0.23^a^		2.12 ± 0.05^a^	1.00 ± 0.27^a^	2.45 ± 0.60^a^

**Two-way ANOVA: *p* values**									

Stocking density		<0.05		<0.05	<0.05		<0.05	>0.05	>0.05
Vitamin C		<0.05		<0.05	>0.05		>0.05	>0.05	>0.05
Interaction		<0.05		<0.05	>0.05		>0.05	>0.05	>0.05

*Note: n* = 3. Overall mean value having different superscript in the same column shows a significant difference (*p* < 0.05; mean ± S.E.); alphabets denote the significant differences between different treatments.

**Table 4:** Effects of stocking density and dietary vitamin C on stress parameters in *Labeo gonius* reared in biofloc system.

Treatments		Parameters						
Stocking density (nos. 50 L^−1^)	Vit C (mg kg^−1^)	Glucose (mg dL^−1^)	SOD (U mg protein^−1^ min^−1^)	MDA (n moles mg protein^−1^)	GST (U mg protein^−1^ min^−1^)	GOT (U L^−1^)	GPT (U L^−1^)	Cortisol (ng L^−1^)
5	0	35.97 ± 0.22^a^	60.6 ± 0.90^ab^	9.95 ± 0.03^d^	8.7 ± 0.15^a^	35.5 ± 1.5^a^	15.89 ± 0.82^a^	3.37 ± 0.001^c^
	500	39.96 ± 0.40^b^	54.3 ± 0.364^a^	6.3 ± 0.15^a^	9.9 ± 0.44^b^	36.9 ± 1^a^	16.46 ± 0.35^a^	3.18 ± 0.002^ab^
	1000	38.5 ± 0.16^ab^	60.2 ± 1.39^ab^	8.27 ± 0.09^b^	8.9 ± 0.12^ab^	39.91 ± 1^a^	16.00 ± 0.88^a^	3.31 ± 0.003^bc^

10	0	41.04 ± 0.71^b^	54 ± 1.51^a^	11.4 ± 0.12^e^	16.5 ± 0.15^d^	39.7 ± 1^a^	16.82 ± 4.12^a^	3.27 ± 0.001^bc^
	500	40.2 ± 0.29^b^	64.4 ± 5.34^b^	9.67 ± 0.17^cd^	15.4 ± 0.15^c^	37.14 ± 1.1^a^	16.14 ± 3.30^a^	3.13 ± 0.006^a^
	1000	41.84 ± 0.31^bc^	63.6 ± 1.52^b^	10.41 ± 0.06^d^	15.5 ± 0.29^cd^	36.13 ± 1^a^	16.9 ± 3.24^a^	3.12 ± 0.003^a^

15	0	49.13 ± 0.52^e^	58.5 ± 1.49^ab^	15.4 ± 0.36^g^	20.6 ± 0.12^f^	38.53 ± 0.4^a^	17.5 ± 2.02^a^	5.47 ± 0.088^f^
	500	45.28 ± 0.01^cd^	62.1 ± 1.85^b^	8.97 ± 0.09^bc^	18.6 ± 0.15^e^	35.53 ± 1.8^a^	16.22 ± 1.47^a^	4.17 ± 0.033^d^
	1000	45.79 ± 1.79^de^	57.2 ± 1.62^ab^	13.63 ± 0.26^f^	18.2 ± 0.15^e^	37.70 ± 1.2^a^	15.61 ± 2.02^a^	4.66 ± 0.081^e^

**Two-way ANOVA (*p* values)**								

Stocking density		<0.05	>0.05	<0.05	<0.05	>0.05	>0.05	<0.05
Vit C		>0.05	>0.05	<0.05	<0.05	>0.05	>0.05	<0.05
Interaction		<0.05	<0.05	<0.05	<0.05	<0.05	>0.05	<0.05

*Note: n* = 3. Overall mean value having different superscript in the same column shows a significant difference (*p* < 0.05; mean ± S.E.); alphabets denote the significant differences between different treatments.

## Data Availability

The data that support the findings of this study are accessible upon reasonable request from the corresponding author.
